# Secreted parasite Pin1 isomerase stabilizes host PKM2 to reprogram host cell metabolism

**DOI:** 10.1038/s42003-019-0386-6

**Published:** 2019-04-30

**Authors:** Justine Marsolier, Martine Perichon, Jonathan B. Weitzman, Souhila Medjkane

**Affiliations:** 1 Sorbonne Paris Cité, Epigenetics and Cell Fate, Université Paris Diderot, CNRS, UMR 7216 Paris, France; 2 Present Address: Institut Curie, 26 rue d′Ulm, 75005 Paris, France

**Keywords:** Cancer metabolism, Cell signalling

## Abstract

Metabolic reprogramming is an important feature of host–pathogen interactions and a hallmark of tumorigenesis. The intracellular apicomplexa parasite *Theileria* induces a Warburg-like effect in host leukocytes by hijacking signaling machineries, epigenetic regulators and transcriptional programs to create a transformed cell state. The molecular mechanisms underlying host cell transformation are unclear. Here we show that a parasite-encoded prolyl-isomerase, TaPin1, stabilizes host pyruvate kinase isoform M2 (PKM2) leading to HIF-1α-dependent regulation of metabolic enzymes, glucose uptake and transformed phenotypes in parasite-infected cells. Our results provide a direct molecular link between the secreted parasite TaPin1 protein and host gene expression programs. This study demonstrates the importance of prolyl isomerization in the parasite manipulation of host metabolism.

## Abstract (web summary)

Marsolier et al. show that a parasite-encoded prolyl-isomerase, TaPin1, stabilizes host pyruvate kinase isoform M2 (PKM2), leading to metabolically transformed phenotypes in parasite-infected cells. This study illustrates how intracellular parasites use prolyl isomerization to manipulate host metabolism to their advantage.

## Introduction

The metabolic switch to aerobic glycolysis is an important characteristic of tumorigenesis and cellular reprogramming^1,2^. Metabolic exchange is also a key factor in parasite–host interactions and the manipulation of host cell phenotypes. Several parasites enter into intricate metabolic exchange with their host cells^3^. *Theileria* parasites are remarkable for their ability to interfere with host signaling pathways, activate nuclear transcription factors (e.g., c-Myc, HIF1α, and AP-1) and transform host leukocytes^4–7^. We previously described a Warburg-like phenotype in infected leukocytes associated with stabilization of hypoxia induced factor 1α (HIF1α) and induction of aerobic glycolytic genes^4,8,9^. We also discovered that *Theileria* parasites secrete a Peptidyl-prolyl isomerase (TaPin1) into the host cell, which induces proliferation via the host transcription factor c-Jun^10^. We found that TaPin1 is targeted by the theilericidal drug Burparvaquone, though there may be additional pathways targeted by this drug. In this study, we set out to identify molecular mechanisms that could link the secreted parasite TaPin1 protein to host signaling pathways. We show that TaPin1 interacts with the host Pyruvate Kinase Isoform M2 (PKM2), leading to its stabilization and subsequent HIF1α-dependent induction of glycolytic enzymes that contribute to host transformed phenotypes.

## Results

### Parasite TaPin1 stabilizes host PKM2 protein

**Fig. 1:**
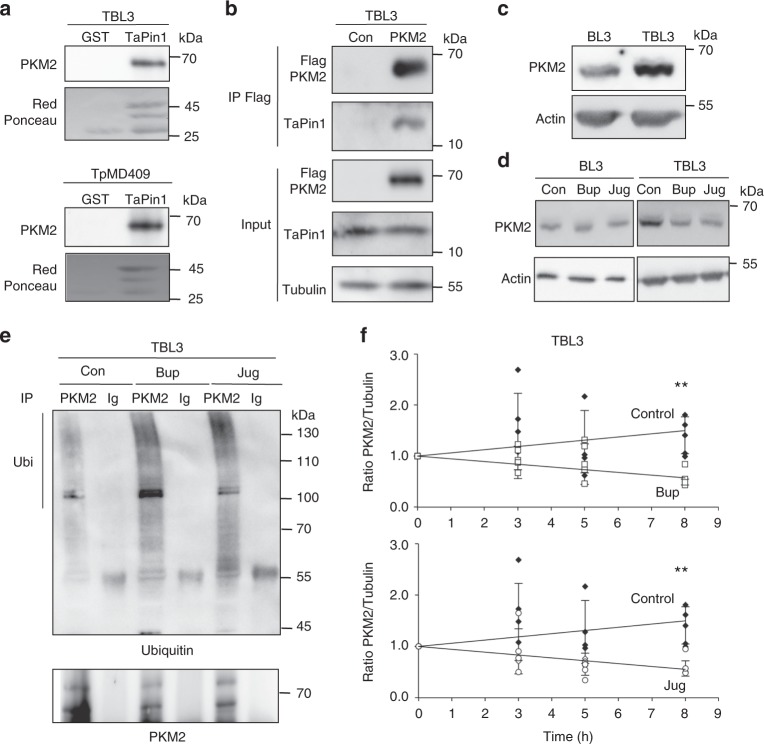
Parasite TaPin1 stabilizes host PKM2 protein. **a** Recombinant GST-TaPin1 protein interacted with endogenous bovine PKM2 protein in whole-cell lysates from lymphocyte cell lines infected with *T. annulata* (TBL3) or *T. parva* (TpMD409). Original blot images are shown in Supplementary Fig. 7a. **b** Flag-PKM2 interacted with endogenous TaPin1 protein in infected TBL3 cells. Flag-PKM2 or Flag-Control [Con] were immunoprecipitated (IP), followed by immunoblot analysis with indicated antibodies. Original blot images are shown in Supplementary Fig. 7b. **c** Bovine PKM2 protein expression in uninfected BL3 and infected TBL3 cells (bovine Beta-actin was a loading control). Original blot images are shown in Supplementary Fig. 7c. **d** TaPin1 inhibition by Buparvaquone [Bup] or Juglone [Jug] decreased host PKM2 protein levels compared to untreated control [Con] in *T. annulata* infected TBL3 cells but had no effect on uninfected BL3 cells. Original blot images are shown in Supplementary Fig. 7d. **e** Buparvaquone [Bup] or Juglone [Jug] treatment increased host PKM2 protein ubiquitination in infected cells. Infected cells were incubated with the proteasome inhibitor MG132 for 3 h in the presence of Buparvaquone [Bup], or Juglone [Jug] or no inhibitor [Con]. Cell extracts were immunoprecipated [IP] using antibodies against PKM2 or controls [Ig], followed by immunoblot analysis. **f** TaPin1 Iinhibition decreased the half-life of endogenous PKM2 protein. TBL3 cells were incubated with cycloheximide and Bup or Jug, followed by immunoblot analysis with a PKM2 antibody and quantification compared to tubulin expression. Data represent four independent experiments (average ± sd). The *p*-values were calculated using the Dunnett test for multiple comparisons with the control conditions. **p < 0.01

To search for Pin1 interactors, we expressed ectopic, tagged Pin1 in fibroblasts and performed immunoprecipitation followed by mass spectrometry analysis (Supplementary Fig. 1a). We identified several potential interacting proteins in the cytoplasm. This list of interacting proteins is unlikely to be exhaustive, as the previously identified FBW7 protein was not found in this screen^11^. One of the most abundant Pin1-interactors was PKM2 (Supplementary Data 1). We investigated whether GST-TaPin1 could also interact with host PKM2 in extracts from bovine leukocyte cell lines infected with either *T. annulata* or *T. parva*. (Fig. 1a). To confirm this interaction, we transfected Flag-tagged PKM2 into TBL3 infected cells and showed that it interacts with the endogenous parasite TaPin1 protein (Fig. 1b). To examine the consequences of the TaPin1–PKM2 interaction, we monitored the levels of the endogenous PKM2 protein in parasite-infected TBL3 cells, compared to non-infected BL3 cells (Fig. 1c) and observed elevated levels in parasitized cells. Furthermore, treatment with two pharmacological PPIase inhibitors, Buparvaquone or Juglone^10^, led to a reduction in the PKM2 protein levels in TBL3 parasitized cells (Fig. 1d and quantification in Supplementary Fig. 1b). Treatment with Buparvaquone or Juglone had no effect on the levels of *PKM2* mRNA in parasitized TBL3 cells (Supplementary Fig. 1c). Inhibition of TaPin1 with Buparvaquone or Juglone or ectopic expression of TaPin1 did not change basal PKM2 protein levels in control BL3 cells (Supplementary Fig. 1b, d). It could be that BL3 cells lack effectors required for the TaPin1 effects. To test whether parasite TaPin1 could regulate bovine PKM2 protein stability, we investigated PKM2 ubiquitination and half-life. We found that Buparvaquone/Juglone treatment induced the ubiquitination of PKM2 (Fig. 1e) and reduced the half-life of PKM2 in parasitized TBL3 cells (Fig. 1f and Supplementary Fig. 1e) as measured by cycloheximide pulse-chase experiments. Together these results showed that the *Theileria* parasite TaPin1 prolyl isomerase interacts (directly or indirectly) with host bovine PKM2 and leads to its stabilization.

### Parasite TaPin1 regulates host cell metabolism

**Fig. 2:**
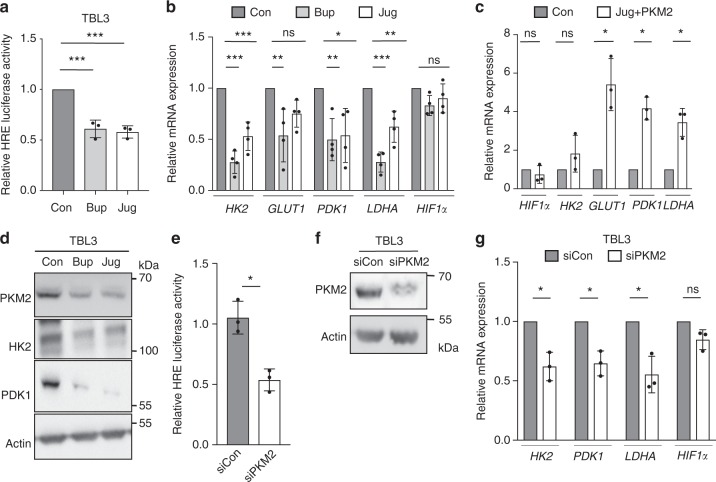
Parasite TaPin1 regulates host HIF1α activity and metabolic gene expression. **a** TaPin1 inhibition with Buparvaquone/Juglone treatment decreased the activity of a 5×HRE HIF1α Luciferase reporter. **b** TaPin1 inhibition decreased the expression of HIF1α target genes in TBL3 cells. qPCR analysis of host gene expression upon TaPin1 inhibition by Buparvaquone or Juglone compared to untreated controls [Con]. Bovine Beta*-*actin and H2A mRNAs were used for normalization. **c** Ectopic expression of PKM2 in parasite-infected TBL3 cells rescued the repression of host glycolytic enzymes observed upon TaPin1 inhibition with Juglone treatment. **d** TaPin1 inhibition decreased the expression of glycolytic enzyme proteins. (Bovine Beta-actin was a loading control). Original blot images are shown in Supplementary Fig. 7e. **e** siPKM2 decreased HIF1α activity on 5xHRE Luciferase reporter. **f** Efficiency of siPKM2. Bovine Beta-actin was used as a loading control. Original blot images are shown in Supplementary Fig. 7f. **g** PKM2 knockdown decreased the mRNA levels of the HIF1α targets (siPKM2 or siControl). Bovine Beta-actin and H2A mRNAs were used for normalization. Data represent three independent experiments (average ± sd). The *p*-values were calculated using the Dunnett test for multiple comparisons (Fig. 2a, b). The *p*-values were calculated using the Mann–Whitney test (Fig. 2c–f). *p < 0.05, **p < 0.01, ***p < 0.001

In addition to its role in phosphoenolpyruvate phosphorylation, PKM2 acts as a cofactor for HIF1α, a transcription factor critical for the Warburg effect and the transcription of glycolytic enzymes in cancer cells^12–14^. We tested whether stabilization of host PKM2 by the parasite TaPin1 protein could affect HIF1α functions. We observed that TaPin1 inhibition (via Buparvaquone or Juglone treatment) reduced transcriptional activity of HIF1α (40–50%), measured by a hypoxia-responsive element (5×HRE) Luciferase reporter (Fig. 2a). The reduced HIF1α activity correlated with reduced expression of HIF1α-target genes linked to host cell metabolism, namely genes encoding the glycolytic enzymes Hexokinase 2 [*HKII*], the Glucose transporter 1 [*GLUT1*], Pyruvate dehydrogenase kinase [*PDK1*] and Lactate dehydrogenase [*LDHA*]. The expression of HIF1α-target genes was reduced at the mRNA level (40–70%) (Fig. 2b) and protein levels (Fig. 2d). The PPIase inhibitors had no effect on the expression of *HIF1α* transcripts (Fig. 2b). Experiments in control BL3 cells indicated that Buparvaquone or Juglone treatment did not affect the expression of glycolytic enzymes in unparasitized cells (Supplementary Fig. 2a). To show that the regulation of metabolic enzymes could be via parasite TaPin1-dependent stabilization of host PKM2 protein, we transfected exogenous PKM2 into TBL3 cells prior to treatment with TaPin1 inhibitors. The forced expression of PKM2 rescued expression of the metabolic enzymes (GLUT1, LDHA, and PDK1) in the presence of Juglone inhibitor (Fig. 2c). Conversely, siRNA silencing of endogenous, bovine *PKM2* reduced HIF1α-activity using the 5xHRE luciferase reporter assay in TBL3 cells (Fig. 2e, f). This correlated with a marked reduction in the expression of host glycolytic enzymes without affecting the levels of HIF1α transcripts in TBL3 cells (Fig. 2g). Notably, siRNA directed against *PKM2* did not decrease the expression of the glycolytic enzymes in control BL3 cells (Supplementary Fig. 2b, c).

### Parasite TaPin1 can partially rescue PKM2 regulation

**Fig. 3:**
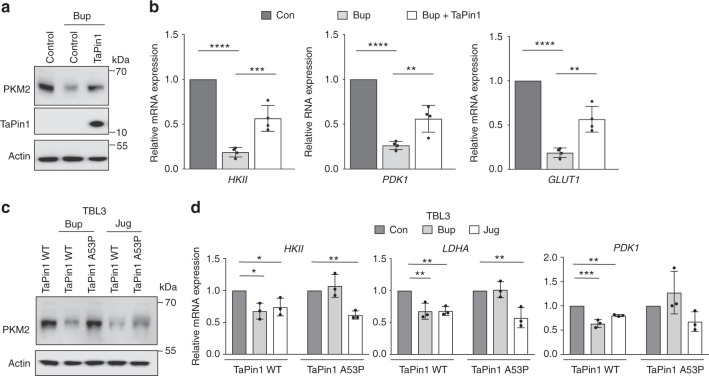
Parasite TaPin1 can partially rescue PKM2 regulation and glycolytic gene expression in TBL3 cells. **a** Flagged-TaPin1 could partially rescue decreased PKM2 protein levels upon Buparvaquone treatment. Bovine Beta-actin was the loading control. Original blot images are shown in Supplementary Fig. 7g. **b** TaPin1 could partially rescue the expression of HIF1α target genes upon Buparvaquone treatment. Bovine Beta-actin was used for normalization. **c** Mutant TaPin1-A53P, but not WT TaPin1, is resistant to Buparvaquone effects on PKM2 protein expression in *T. annulata* infected TBL3 cells. Bovine Beta -actin was the loading control. **d** Analysis of bovine glycolytic enzyme gene expression by qPCR in TBL3 cells following Buparvaquone [Bup] or Juglone [Jug] treatment and simultaneous transfection of TaPin1 WT or Mutant A53P. Bovine Beta *-*actin mRNAs was used for normalization. Original blot images are shown in Supplementary Fig. 7h. Data represent three or four (Fig. 3b) independent experiments (average ± sd). The *p*-values were calculated using the Bonferroni test for multiple comparisons (Fig. 2b) and Dunnett test for multiple comparisons to the control (Fig. 2d). *Con*, control. **p < 0.01, ***p < 0.001, ****p < 0.0001

To provide further support for a role of TaPin1-PKM2 in the regulation of host metabolic enzymes, we tested TaPin1’s ability to rescue the Buparvaquone effects. Overexpression of TaPin1 could partially rescue the expression of PKM2 proteins in TBL3 cells treated with Buparvaquone (Fig. 3a) leading to a partial rescue of the expression of glycolytic enzymes (Fig. 3b). To demonstrate the importance of the prolyl isomerase activity, we tested TaPin1-K38A and TaPin1-S42E catalytic mutants^10,15^. These failed to rescue PKM2 stabilization or glycolytic enzyme expression (Supplementary Fig. 3a, b). We previously reported a mutation in the parasite *TaPin1* gene that resulted in a Buparvaquone-resistant protein^10^. Interestingly, transfection experiments in TBL3 cells showed that the TaPin1-A53P mutant was not sensitive to Buparvaquone effects on PKM2 protein levels (Fig. 3c) and the expression of the glycolytic enzymes in TBL3 cells (Fig. 3d). The TaPin1-A53P mutant remains sensitive to the Juglone drug^10^ and failed to maintain PKM2 protein levels in Juglone-treated TBL3 cells (Fig. 3c) or glycolytic enzyme expression (Fig. 3d). Finally, we performed knockdown experiments to exclude a role for the endogenous bovine Pin1 protein in regulating PKM2 and metabolic enzymes in TBL3 cells. We tested siRNA knockdown of endogenous bovine *Pin1* and showed that siBtPin1 did not affect PKM2 protein expression (Supplementary Fig. 4a) or HIF1α-target gene expression (Supplementary Fig. 4b). These combined data support a role for the secreted TaPin1 protein in regulating host gene expression through prolyl isomerase stabilization of host PKM2 and the HIF1α pathway.

### The TaPin1–PKM2 axis is important for glucose uptake and host cell transformation

**Fig. 4:**
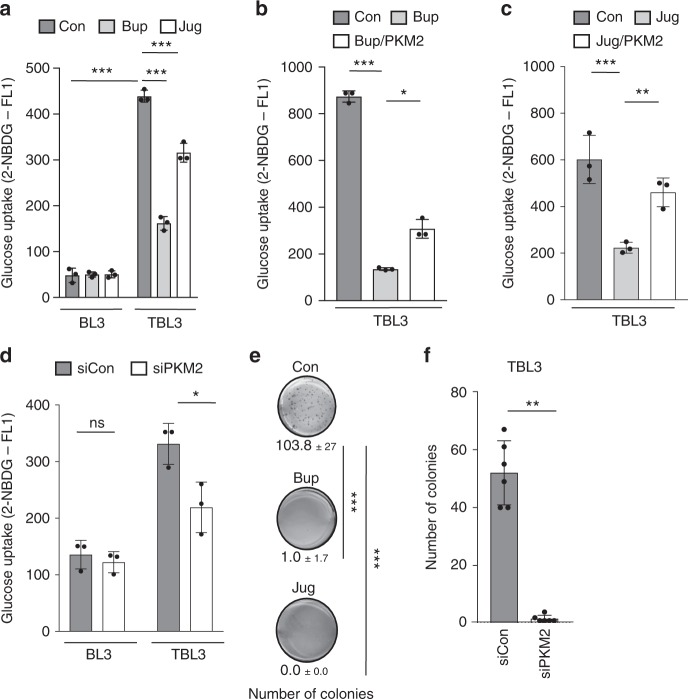
The TaPin1-PKM2 axis is important for glucose uptake and transformation. **a** Glucose uptake (fluorescence intensity) decreased in *Theileria*-infected TBL3 cells upon TaPin1 inhibition by Buparvaquone or Juglone drugs. **b**, **c** Ectopic bovine PKM2 partially rescued glucose uptake following TaPin1 inhibition with Buparvaquone (**b**) or Juglone (**c**) in TBL3 cells. **d** PKM2 knockdown reduced glucose uptake in parasitized TBL3 cells (Fluorescence intensity). **e** Inhibition of TaPin1 by Buparvaquone or Juglone affected TBL3 colony formation in soft-agar. **f** siPKM2 reduced TBL3 cell colony formation in soft agar. Data represent three independent experiments (average ± sd). The *p*-values were calculated using the Bonferroni test for multiple comparisons (**a**–**e**) and the Mann–Whitney test (**f**). *p < 0.05, **p < 0.01, ***p < 0.001

To test the functional significance of TaPin1–PKM2 signaling on host transformation, we measured the effects of TaPin1 inhibitors on glucose uptake in parasitized cells. Parasitized TBL3 cells exhibited a 9-fold increase in glucose uptake compared to control BL3 cells (Fig. 4a). This parasite-induced effect was reduced by treatment with Juglone or Buparvaquone (Fig. 4a) with no effect in BL3 cells. As demonstrated above for PKM2 stabilization and glycolytic enzyme expression, the Buparvaquone and Juglone effects on glucose uptake could be partially rescued by overexpression of ectopic PKM2 in TBL3 cells (Fig. 4b, c). Furthermore, knockdown of endogenous PKM2 in parasitized cells also led to a reduction in glucose uptake (Fig. 4d). Notably, the overexpression of ectopic PKM2 or siRNA-mediated knockdown PKM2 did not affect the glucose uptake of control BL3 cells (Supplementary Fig. 2d and Fig. 4d). Finally, we tested the contribution of TaPin1 and PKM2 to the transformed phenotype of parasitized cells. Treatment of parasite-infected TBL3 cells with TaPin1 inhibitors, Juglone or Buparvaquone, or knockdown of endogenous *PKM2*, each led to a marked decrease in cell proliferation (Supplementary Fig. 5a, b) and the growth of colonies in soft agar, an effective in vitro assay for transformation (Fig. 4e, f). Once again, siPin1 knockdown of bovine *Pin1* did not affect colony growth of TBL3 cells (Supplementary Fig. 4c). These combined experiments suggest that the stabilization of host bovine PKM2 protein by the secreted parasite TaPin1 PPIase leads to activation of HIF1α-dependent metabolic genes that are essential for glucose metabolism and host cell transformation.

## Discussion

Metabolic reprogramming is a hallmark of cancer cells and is critical for tumor cell survival and proliferation^2,16^. Previous reports of Warburg-like “aerobic glycolysis” in bovine leukocyte cell lines transformed by *Theileria* parasites^17,18^ lacked a direct molecular link between intracellular parasites and host metabolic gene expression. Here, we identified the TaPin1–PKM2-HIF1α axis as an integrator of parasite–host interaction. We showed that TaPin1 and bovine PKM2 interact (either directly or indirectly) and that this interaction leads to PKM2 stabilization and HIF1α-dependent glycolytic enzyme expression.

PKM2 plays a critical role in the metabolic rewiring that underlies tumorigenesis^19^. Non-canonical PKM2 functions include nuclear transcriptional regulation^12–14,20^ and enhanced HIF1α binding to HRE in metabolic genes^13,14^. The PKM2 protein is finely tuned by cancer cells, involving mRNA splicing, ERK-dependent phosphorylation, and subcellular relocalization. We propose prolyl isomerization and protein stabilization as another level of regulation that parasites exploit to maintain a Warburg-like phenotype. These data add to the descriptions of the complex metabolic relationships between parasites and their host cells^3^. Increased glucose flux and energy metabolism could support the biosynthetic requirements of both the parasite and the hyperproliferating host cell. The secreted parasite TaPin1 protein could activate multiple pathways that are important for host cell proliferation and metabolism. For example, TaPin1 stabilizes the host transcription factor c-Jun by regulating a ubiquitin ligase, FBW7^10^. Additional secreted proteins may contribute to subverting the host cell and perhaps TaPin1 has additional targets. Here, we show that TaPin1 also activates HIF1α-regulated target genes via stabilization of PKM2. These results provide a molecular link between prolyl isomerization and metabolic manipulation by intracellular parasites.

## Methods

### Cell lines and culture conditions

All infected bovine cell lines were previously described: the TBL3 cell line was derived from in vitro infection of the spontaneous bovine-B lymphosarcoma cell line, BL3, with Hissar stock of *T.annulata*. The TpMD409 lymphocyte cell line is infected with *T. parva*. The culture conditions of these cell lines were described previously^10^. Parasite-infected cell lines were provided by the Langsley laboratory. Cells were cultured in a humidified 5% CO_2_ atmosphere at 37 °C in RPMI 1640 (Gibco-BRL), supplemented with 4 mM l-Glutamine, 25 mM HEPES, 10 µM Beta-mercaptoethanol, 10% heat-inactivated Fetal calf serum and 100 µg/ml penicillin/streptomycin. All cell lines were mycoplasma negatives. The anti-parasite drug Buparvaquone (BW720c) was used for 72 h at 200 ng/ml (Chemos GmbH, Ref: 88426-33-9) (Supplementary Fig. 5a, b). Cells were treated 72 h with Juglone at 5 µM resuspended in Ethanol (Sigma, Ref: H47003).

### Plasmids and transfection

Bovine PKM2 (NM_001205727/NP_001192656.1) was cloned between restriction sites *HindIII* and *EcoRI* in p3xFlag-myc-CMV-24 using the following oligonucleotides: Fwd—cccaagctttcgaagcaccacagcgacg and Rev—CGGAATTCGAtggcacaggaactacacg. Parasite gene TaPin1 (TA18945) was cloned between restriction sites *Xho*I and *Not*I in pREV-HA-Flag-RIL2 using oligonucleotides: Fwd—CCGCTCGAGGCCCACTTGCTACTAAAG and Rev—ATAAGAATGCGGCCGCTTATGCGATTCTATATATAAGATG. Point mutations TaPin1 K38A, A53P^10^ and S47E were created from pRev-HA-Flag-TaPin1 WT-RIL2 using a set of primers following a 3-step PCR using the following oligos:

**Table:** 

Name	Sequence Fwd 5′-3′	Sequence Rev 5′-3′
HsPin1	CCGCTCGAGGCGGACGAGGAGAAGCTG	AAGGAAAAAAGCGGCCGCTCACTCAGTGCGGAGGATGA
TaPin1	CCGCTCGAGGCCCACTTGCTACTAAAG	ATAAGAATGCGGCCGCTTATGCGATTCTATATATAAGATG
TaPin1 K38A	GCCCACTTGCTACTAGCGCACACTGGATCTAGG	CCTAGATCCAGTGTGCGTAGTAGCAAGTGGGC
TaPin1 S47E	CTAAAGCACACTGGAGAGAGGAATCCAGTGAATAG	CTATTCACTGGATTCCTCTCTCCAGTGTGCTTTAG
TaPin1 GST	CGCGGATCCGCCCACTTGCTACTAAAG	CCGGAATTCTTATGCGATTCTATATATAAGATG
PKM2 bov	CCCAAGCTTTCGAAGCACCACAGCGACG	CGGAATTCGATGGCACAGGAACTACACG

BL3 and TBL3 cells were transfected with indicated plasmids using Neon Transfection kit (Invitrogen)^10^. Cells were single or double transfected with 400 nM of siRNA. Ectopic PKM2, TaPin1 WT and mutants were transfected between 24 and 36 h after drug treatment.

**Table:** 

Name	Sequence Fwd 5′-3′
BtPKM2	TTGTTCGAGGAGCTCCGTCG
BtPin1	GCCATTTGAAGACGCCTCC

### RNA extraction and reverse transcription-qPCR

Total cellular RNAs were extracted using a NucleoSpin RNA Kit (Macherey Nagel, Ref: 740955). cDNA synthesis was performed using the Reverse Transcriptase Superscript III (Invitrogen, Ref: 18080051). Quantitative PCR amplification was performed in the ABI 7500 machine (Applied Biosystems) using the Sybr Green reagent (Applied Biosystems, Ref: 4309155). The detection of a single product was validated by dissociation curve analysis. The bovine Beta-actin and H2A qPCR were used for normalization. Relative quantities of mRNA were analyzed using the delta Ct method.

**Table:** 

Name	Sequence Fwd 5′-3′	Sequence Rev 5′-3′
HKII	CGACCAAGTGCAGAAGGTTG	CGTCTGGAGTAGACCTCAC
GLUT1	CATGGAGCCCACCAGCAAG	CGTTGATGACTCCAGTGTTG
LDHA	GGCTACACATCCTGGGCC	GGAACACTAAGGAAGACATCC
PDK1	CTAGGCGTCTGTGTGATTTG	GATAGAGGTGGGATGGTAC
HIF1a	CATGTGACCACGAGGAAATG	TAGTTCTCCCCCGGCTAGTT
PKM2	CCATTACCAGCGACCCCAC	GTATCTGGCCACCTGATGTG
H2A	GTCGTGGCAAGCAAGGAG	GATCCGGCCGTTAGGTACTC
BETA‐ACTIN	GGCATCCTGACCCTCAAGTA	CACACGGAGCTCGTTGTAGA

### Immunoblot analysis and immunostaining

Total proteins were extracted with Laemmli lysis buffer. Samples were sonicated: 30 s ON/30 s OFF for 5 min, and then resolved on 10.5% acrylamide/bis-acrylamide SDS-PAGE gels and transferred to nitrocellulose membranes (Thermo Fisher Scientific, MA, USA) in transfer buffer. Protein transfer was assessed by performing a Ponceau-red staining. Membranes were then blocked for 1 h at room temperature in Tris-buffered saline pH 7.4 containing 0.1% Tween-20 and 5% milk. Incubations with primary antibodies were performed at 4 °C overnight using antibody dilutions as manufacturer recommendations in Tris-buffered saline pH 7.4, 0.05% Tween-20 and 5% milk. Proteins were detected by chemiluminescence (Thermo Fisher Scientific) following the manufacturer’s instructions following 1 h incubation at room temperature with an anti-rabbit or anti-mouse peroxidase-conjugated secondary antibody (Jackson ImmunoResearch, Ref: 111-035-003 or Ref: 115-035-003). We used these antibodies: Rabbit anti-TaPin1 (Home-made antibody, Proteogenix, Peptide used: NPVNRNTGMAVTR-Cys—Dilution 1/100), mouse anti-αTubulin (Sigma, Ref: T9026—Dilution 1/1000), rabbit anti-PDK1 (C47H1n Cell signaling—Dilution 1/500), rabbit anti-Hexokinase II (Cell Signaling, Ref: C64G5—Dilution 1/500), rabbit anti-PKM2 (Abcam, Ref: ab38237—Dilution 1/250), mouse anti- Beta-actin (Sigma, Ref: A1978—Dilution 1/1000), mouse anti-ubiquitin (P4D1, Ref: sc-8017 Santa Cruz Biotechnology Inc.—Dilution 1/200) and monoclonal anti-Flag M2-Peroxidase (Sigma, Ref: A8592 – Dilution 1/1000).

### Cycloheximide chase assay

This was performed as previously reported^10^. Briefly, after 72 h of treatment with Buparvaquone or Juglone, infected bovine cell line (TBL3) was treated 30, 60, or 120 min with 100 mg/ml Cycloheximide. Cells were lysed using Laemmli sample buffer, resolved by SDS-PAGE and analyzed by western blot using the indicated antibodies. Relative quantification indicates the PKM2/ Tubulin ratios calculated with Image J software (NIH) and PKM2 levels at time 0 was set as 1. Cycloheximide chase experiments were repeated for four independent biological replicates.

### Viability assays

After plating 1 × 10^4^ cells in 96-well plates in triplicate and Buparvaquone, Juglone was added. After 72 h of treatment, cell viability was measured using the Cell proliferation Kit II–XTT (Roche, measurement of the cellular redox potential) and the GloMax-Multi Detection System (Promega). Cell numbers, as judged by Trypan Blue exclusion test, were determined by counting cells using a Countess automated cell counter (Invitrogen).

### Luciferase assay

Bovine cells were transfected with indicated siRNA and the *HRE* Luciferase reporter, using electroporation (Neon kit, Invitrogen, Ref: MPK1096). Transfection efficiencies were normalized to Renilla activity by co-transfection of the reporter plasmid pRL-TK *Renilla* (Promega, Ref: E6241). Luciferase assays were performed using the Dual-Luciferase Reporter Assay System (Promega, Ref: E1980) in a microplate luminometer, 36 h post-transfection. Relative luminescence was represented as the ratio Firefly/Renilla luminescence, compared with the corresponding empty vector control.

### Soft agar colony forming assay

A two-layer soft agar culture system was performed. A total of 20,000 bovine cells (treated with Buparvaquone or Juglone) or 40,000 bovine cells (transfected with siRNA) were plated in a volume of 1.5 ml (0.7% SeaKem ME Agarose: Lonza, Ref: 50011 + 2× DMEM 20% Fetal calf Serum over 1.5-ml base layer (1% SeaKem ME Agarose + 2× DMEM 20% Fetal calf Serum) in 6-well plates. Cultures were monitored for growth using a microscope. At the time of maximum colony formation (10–15 days in culture), after fixation a staining with 0.005% Crystal Violet (Sigma, Ref: C3886) final colony numbers were counted manually.

### Measurement of glucose uptake

Cells were incubated in media supplemented with the fluorescent d-glucose analog 2-NBDG (2-[N-(7-nitrobenz-2-oxa-1,3-diazol-4-yl) amino]-2-deoxy-d-glucose, Life Technologies, Ref: N13195) at 100 µM for 20 min at 37 °C. After two washing with PBS, cells were analyzed by flow cytometry, fluorescence assessed in FL1 channel. For each measurement, data from 20,000 single cell events was collected (Supplementary Figure 6) using a FACScalibur flow cytometer (Becton Dickinson Immunocytometry Systems).

### GST pull-down

This was performed as previously reported^10^. Briefly, TaPin1 and hPin1 were cloned between restriction sites *BamHI* and *EcoRI* in pGEX-2T plasmid which was kindly provided by G. Del Sal (LNCIB—Laboratorio Nazionale CIB, Trieste, Italy). Plasmid constructs were expressed in *E. coli* strain BL21 and then purified using glutathione-sepharose beads. Concentration of purified protein was estimated using Coomassie blue staining. One microgram of GST fusion proteins coated beads were incubated with 250 µl of cell lysate in 50 mM Tris pH7.6, 150 mM NaCl, 0.1% Triton, for 2 h at 4 °C. Beads were washed five times with 50 mM Tris pH7.6, 300 mM NaCl, 0.5% Triton. Proteins were than revealed by Western Blot analysis using indicated antibodies.

### Complex immunopurification and mass spectrometry analysis

We used retroviral transduction strategy to establish NIH/3T3 cell lines expressing double-tagged proteins. Polyclonal NIH/3T3 cell lines stably expressing Flag-HA-tagged hPin1 or TaPin1 were established. All the proteins were tagged with double-HA (Haemagglutinin) and double-Flag epitopes at the N-terminus. A control cell line transduced with the empty pREV vector was established. We carried out double-affinity purification of Flag-HA-hPin1 from NIH/3T3, using either nuclear soluble or cytoplasmic fractions. Both fractions were then subjected to a two-step immunopurification with Flag and HA antibodies as described previously^21^. Mass spectrometry identification of proteins was carried out in the Taplin Biological Mass Spectrometry Facility (Harvard Medical School, Boston, USA) and the results are shown in Supplementary Data 1.

### Immunoprecipitation—HA

TBL3 transiently expressing PKM2 construct were lysed in the following buffer: 20 mM Tris HCL pH8, 150 mM NaCl, 0.6% NP-40 and 2 mM EDTA. Protein complexes were then affinity-purified on anti-Flag antibody-conjugated agarose (Sigma, Ref: A2220) for bovine lysates. Elution was performed using Flag peptide. After five washes, immunopurified complexes were resolved on 4–12% SDS-PAGE bis-Tris acrylamide gradient gel in MOPS buffer (Invitrogen, Ref: NP 0322 BOX, NP0001-02, respectively).

### Immunoprecipitation—Ubiquitin

This was performed as previously reported^11^. Briefly, after 3 h of treatment with 20 µM MG132 at 37 °C, cells were lysed 10 min on ice in the following buffer: 150 mM NaCl, 1% Nonidet P-40, 0.5% Deoxycholate, 0.1% SDS, 50 mM Tris HCl pH 7.5, 20 mM NEM, 5 mM Iodoacetamide, 100 µM MG132, 2 mg/ml Pefabloc SC (Roche) and 5 µg/ml each Aprotinin, Leupeptin, Pepstatin. Equal amounts of total cellular proteins were immunoprecipitated for 90 min at 4 °C with rabbit Anti-PKM2 (Abcam, Ref: 38237) coupled to protein G sepharose beads (Sigma, Ref: P3296). After three washes, immunoprecipitated proteins were eluted at 95 °C for 5 min in Laemmli sample buffer, resolved by SDS-PAGE and analysed by Western blot using the indicated antibodies. Immunoprecipitation was repeated for three independent biological replicates.

### Data and statistical analysis

The GraphPad PRISM 7 was used for statistics. In all the figures the results represent the mean ± sd of at least three independent experiments. Statistical analysis was performed using the Dunnet for multiple comparisons with the control condition or Bonferroni test for multiple comparisons between samples or Mann–Whitney test for the comparison between two conditions. *p* values of <0.05 were considered statistically significant and are indicated with asterisks *p < 0.05; **p < 0.01; ***p < 0.001, ****p < 0.0001.

### Reporting summary

Further information on experimental design is available in the Nature Research Reporting Summary linked to this article.

## Supplementary information

Reporting Summary

Supplementary Information

Description of Additional Supplementary Files

Supplementary Data 1

## Data Availability

All data generated or analysed during this study are included in this published article, Supplementary Information, and Supplementary Data 1. The list of hPin1 protein interactors identified in this study is included in the Supplementary Data 1. The mass spectrometry proteomics data have been deposited to the ProteomeXchange Consortium via the PRIDE partner repository with the dataset identifier PXD012895.
