# Supplementary material for: Secreted parasite Pin1 isomerase stabilizes host PKM2 to reprogram host cell metabolism
Source: Commun Biol. 2019 Apr 30;2:152. doi: 10.1038/s42003-019-0386-6 (PMC6491484; doi:10.1038/s42003-019-0386-6)
Supplement: Supplementary file 2 — Supplementary Information [file 42003_2019_386_MOESM2_ESM.pdf]

**Supplementary Figure 1**

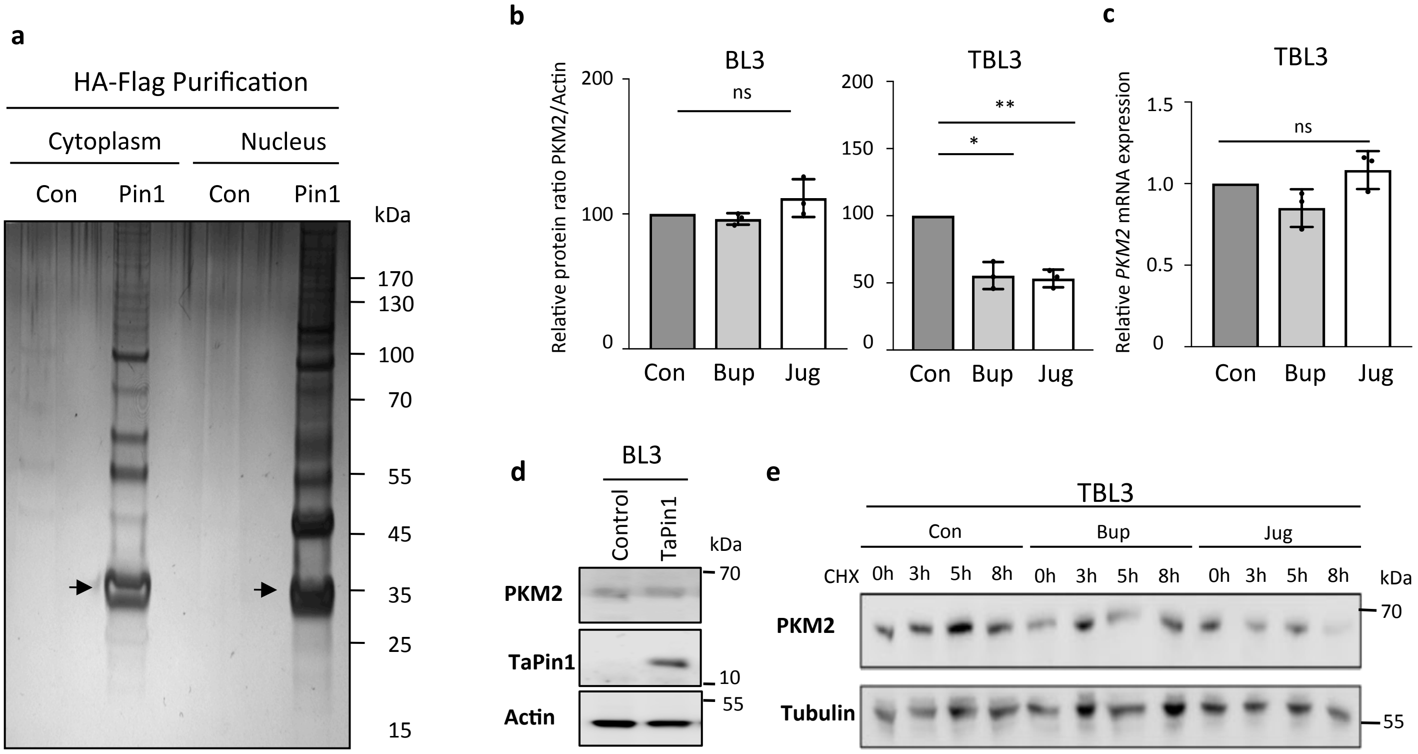


**Supplementary Figure 1: Identification of PKM2 as a Pin1 Partner**

a. Analysis of a double HA-Flag purification of HA-Flag-hPin1 stably expressed in NIH/3T3 cells identified new cytoplasmic and nuclear partners of hPin1 as observed by the silver protein staining. Arrows indicate hPin1 protein.

b. Host PKM2 expression in uninfected BL3 and infected TBL3 cells. Relative quantification showing PKM2/Actin ratios calculated using Image J software.

c. TaPin1 inhibition did not affect the expression of PKM2 in parasite-infected cells. qPCR analysis of PKM2 host gene expression upon TaPin1 inhibition by Buparvaquone [Bup] or Juglone [Jug] compared to untreated controls [Con]. Bovine Beta-actin and H2A mRNAs were used for normalization.

d. Ectopic expression of Flagged-TaPin1 did not change basal PKM2 protein levels in BL3 cells . Bovine Beta-actin was used as loading control normalization.

e. TaPin1 inhibition decreased the half-life of endogenous PKM2 protein. TBL3 cells were incubated with cycloheximide and Buparvaquone or Juglone. Representative immunoblot of PKM2 expression is shown. Bovine tubulin was used as a loading control.

Data in Figure S1b and S1c represent three independent experiments (average ± sd). The p-values were calculated using the Dunnett test for multiple comparisons with the control conditions. *$p < 0.05$, **$p < 0.01$.

## Supplementary Figure 2

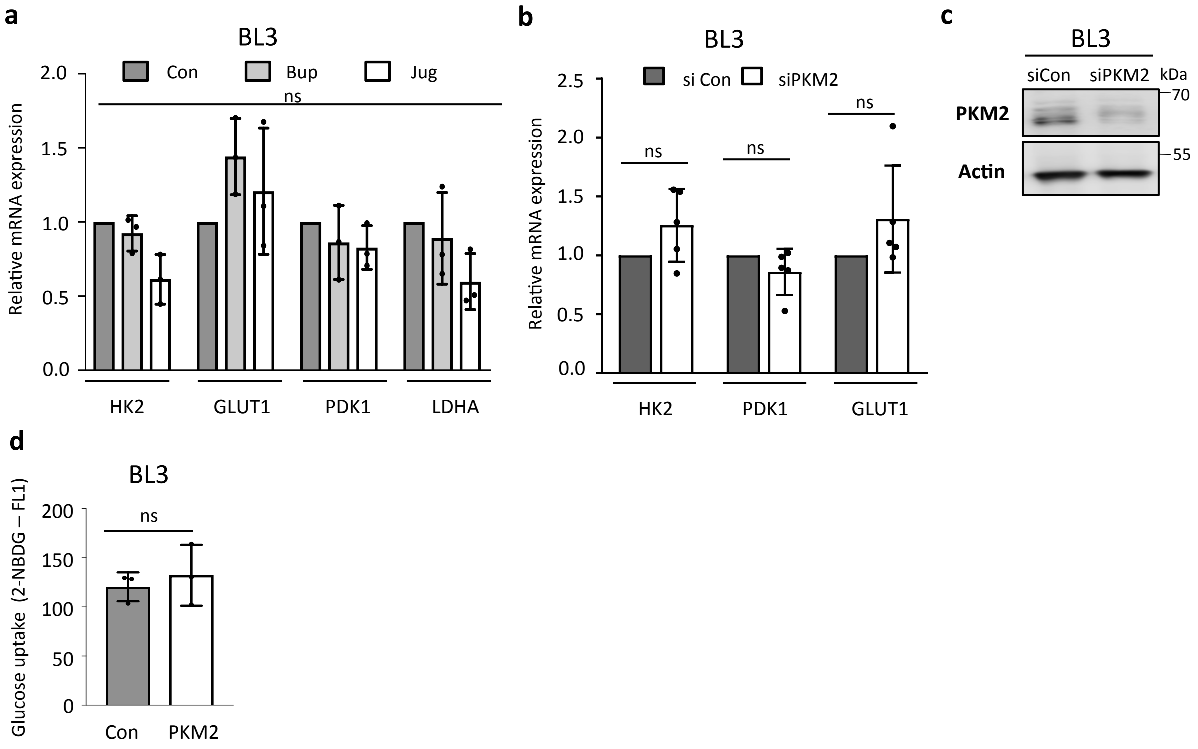


**Supplementary Figure 2 : Modulation of PKM2 does not affect host HIF1α target genes expression and host cell metabolism in BL3 cells.**

a. Buparvaquone or Juglone did not affect the expression of HIF1 α target genes in non-infected BL3 cells. qPCR analysis of host genes expression upon indicated treatment compared to untreated controls [Con]. Bovine Beta-actin and H2A mRNAs were used for normalization.

b. PKM2 silencing did not impact the mRNA levels of the tested HIF1α targets (siPKM2 or siControl) in BL3 cells. Bovine Beta-actin mRNAs was used for normalization.

c. Efficiency of siPKM2. Bovine Beta-actin was used as loading control.

d. Ectopic bovine PKM2 expression did not alter glucose uptake in uninfected BL3 cells (Mean fluorescence intensity, MFI).

Data represent three independent experiments (average ± sd). The p-values in Figure S2a were calculated using the Dunnett test for multiple comparisons with the control conditions. The p-values were calculated using the Mann-Whitney test for Figures S2b and S2d.

## Supplementary Figure 3

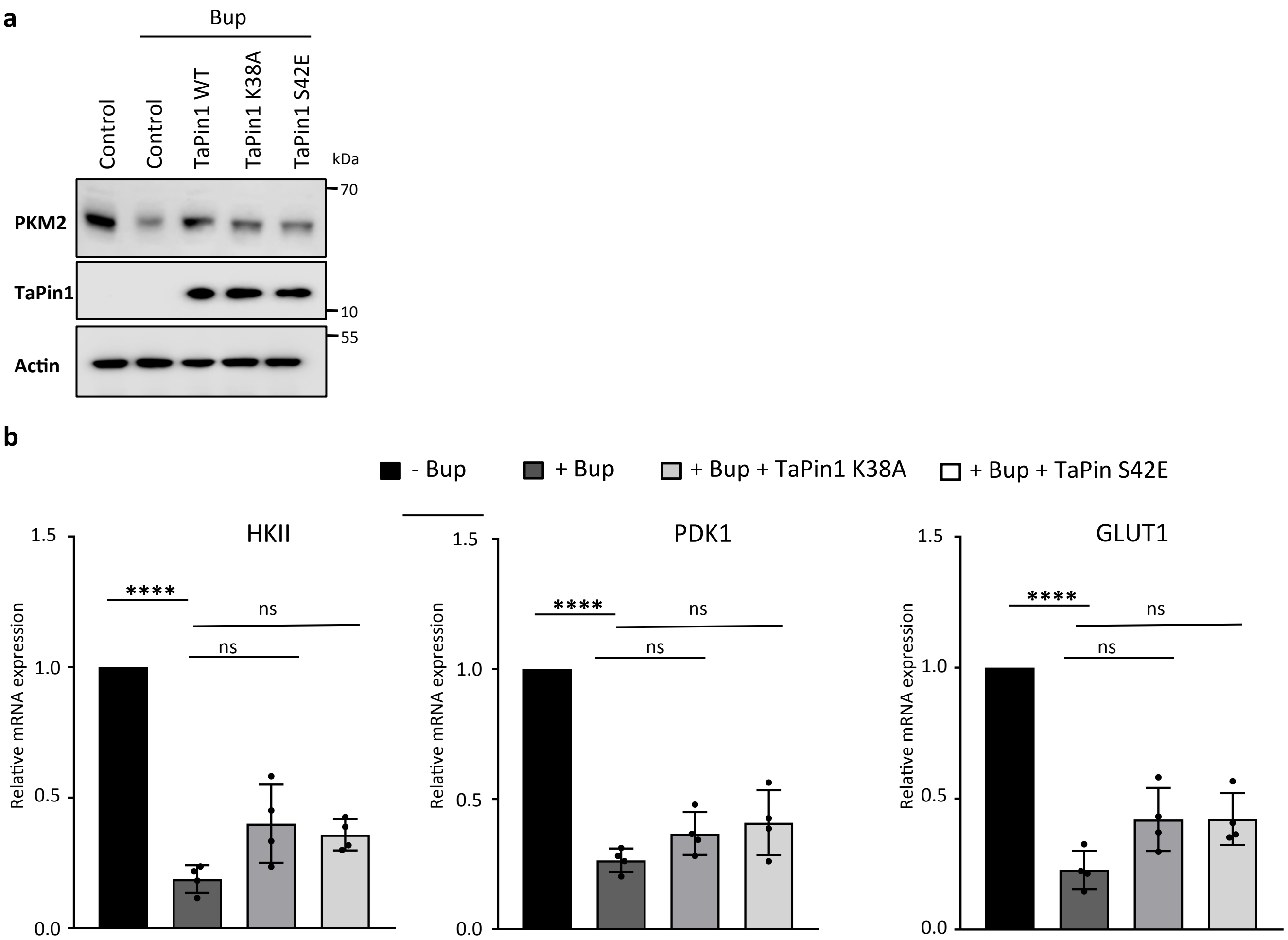


**Supplementary Figure 3: Ectopic expression of TaPin1 and catalytic mutants in TBL3 Buparvaquone treated cells.**

a. Wild-type Flagged-TaPin1, but not the enzyme-dead TaPin1-K38A and TaPin1-S42E, could partially rescue decreased PKM2 protein levels upon Buparvaquone treatment. Bovine Beta-actin was used as loading control.

b. Enzyme-dead TaPin1-K38A and TaPin1-S42E could not rescue decreased HIF1α target genes expression upon Buparvaquone treatment. Bovine Beta-actin was used for normalization.

Data in Figure S3b represent four independent experiments as indicated (average ± sd). The p-values were calculated using the Bonferroni method for multiple comparisons. **$p < 0.01$, ***$p<0.001$, ****$p < 0.0001$.

## Supplementary Figure 4

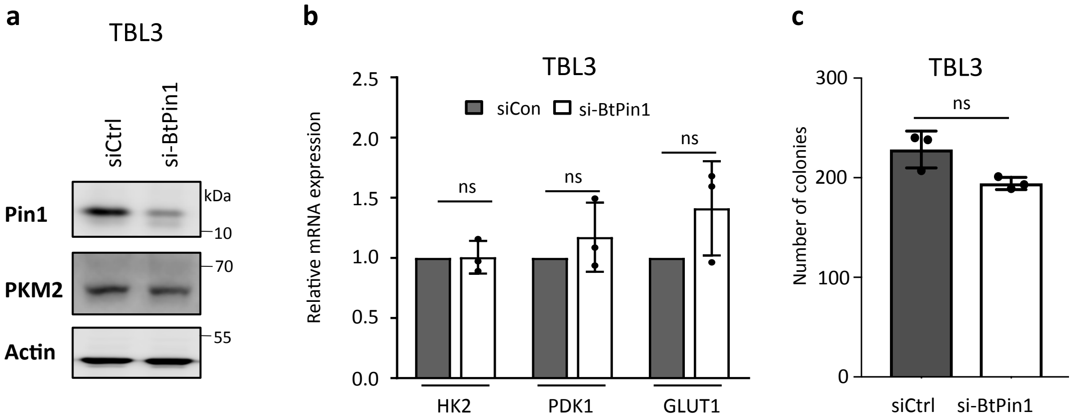


**Supplementary Figure 4: Inhibition of host Pin1 does not affect PKM2 expression nor host HIF1α target gene expression.**

a. Inhibition of host Pin1 (siBtPin1) did not alter PKM2 endogenous protein expression. Bovine Beta-actin was used as loading control.

b. siBtPin1 did not affect the expression of HIF1 α target genes in infected TBL3 cells. qPCR analysis of host genes expression. Bovine Beta-actin was used for normalization.

c. siBtPin1 did not affect TBL3 colony formation in soft-agar.

Data represent three independent experiments (average ± sd). The p-values were calculated using the Mann-Whitney test.

## Supplementary Figure 5

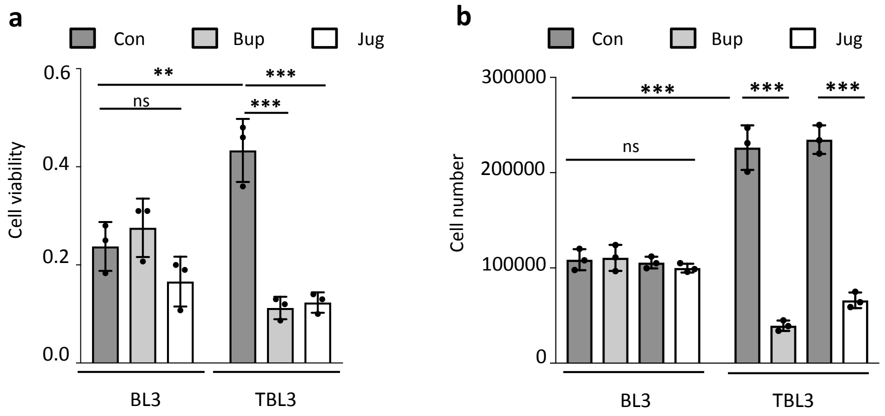


**Supplementary Figure 5: Effect of TaPin1 inhibition on viability of *T. annulata* infected cells.**

a. TaPin1 inhibitors (Buparvaquone or Juglone) decreased the viability of infected TBL3 cells (XTT Cell viability assay at 72h) but not uninfected BL3 cells.

b. TaPin1 inhibitors (Buparvaquone or Juglone) decreased the cell number of infected TBL3 cells after 72h of treatment.

Data represent three independent experiments (average ± sd). The p-values were calculated using the Bonferroni test for multiple comparisons. **p <0.01, ***p <0.001.

**Supplementary Figure 6**

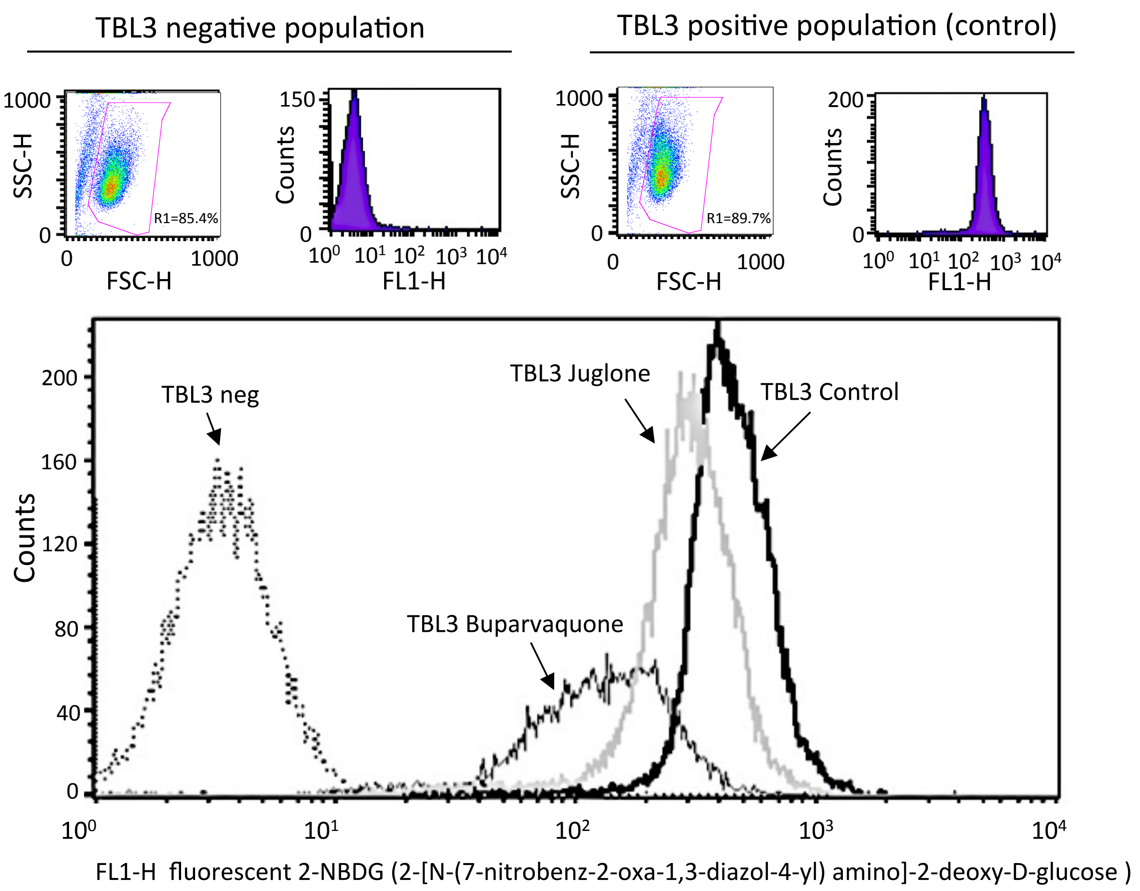


**Supplementary Figure 6 - Figure exemplifying the flow-cytometry gating strategy – Glucose uptake analysis.**

Glucose uptake (FL1-H fluorescence intensity) was measured from R1-gated cells

**Supplementary Figure 7**

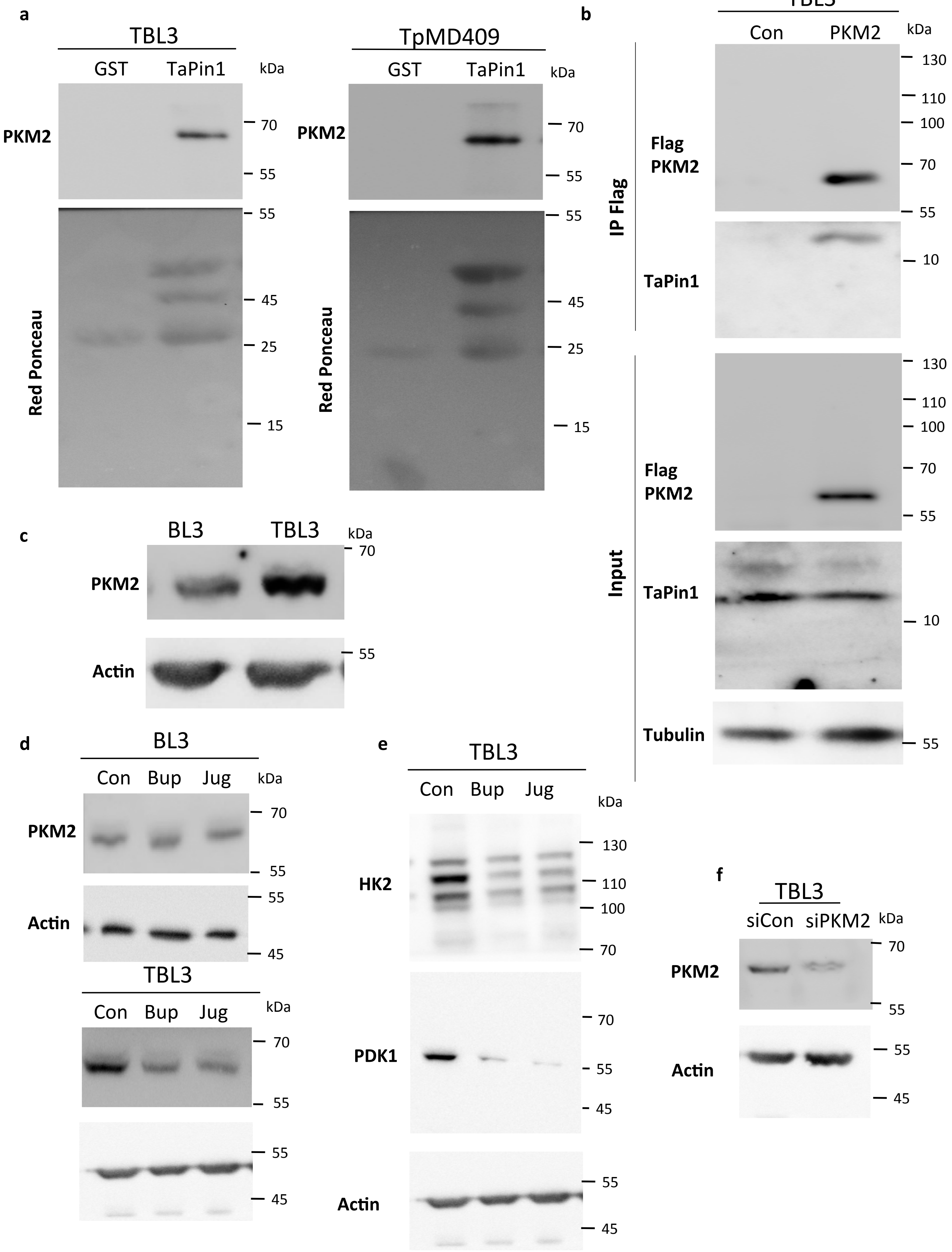

**Supplementary Figure 7**

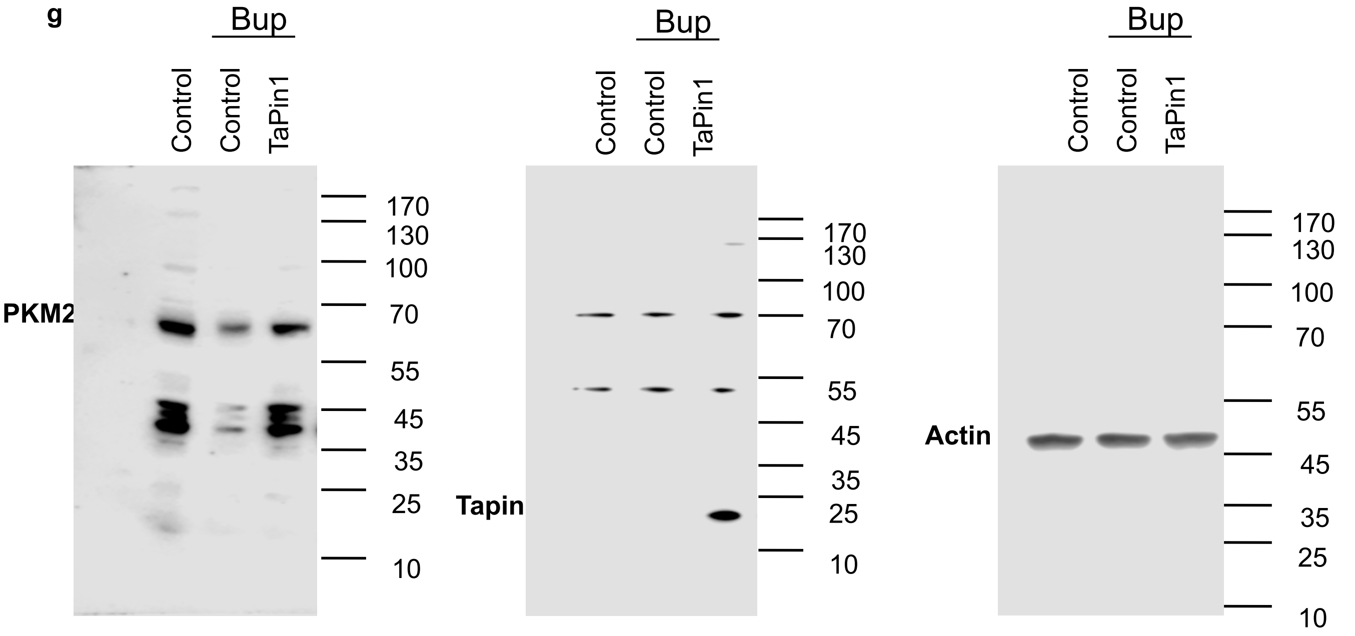


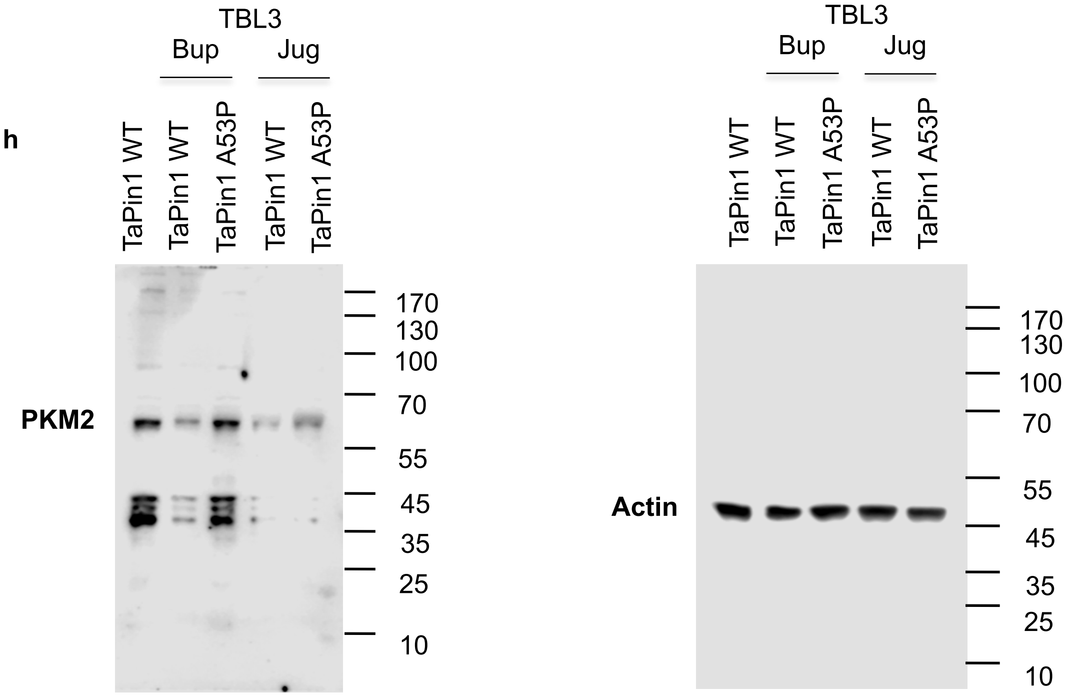


**Supplementary Figure 7: Original blots.**

a. Original blots of Fig.1a. Membranes were cut before incubation with the antibody.

b. Original blots of Fig.1b. Membranes were cut before incubation with the antibody.

c. Original blots of Fig.1c. Membranes were cut before incubation with the antibody.

d. Original blots of Fig.1d. Membranes were cut before incubation with the antibody.

e. Original blots of Fig.2e. Membranes were cut before incubation with the antibody.

f. Original blots of Fig.2f. Membranes were cut before incubation with the antibody.

g. Original blots of Fig.3a.

h. Original blots of Fig.3c.

## Supplementary Methods

### Protein Sequence Analysis by LC-MS/MS

Excised gel bands were cut into approximately 1 $mm^3$ pieces. Gel pieces were then subjected to a modified in-gel trypsin digestion procedure [1]. Solution samples were reduced by using DTT at a 1mM concentration (in 50mM ammonium bicarbonate) for 30 minutes at 60C. The samples are then cooled to room temperature and iodoacetamide (stock in 50mM ammonium bicarbonate) is added to a concentration of 5mM for 15 minutes in the dark at room temperature. DTT was then added to a 5mM concentration to quench the reaction. We then add sequence grade trypsin at a concentration of 5ng/ul. The digestion is over-night at 37C. The samples are then desalted by an in-house made desalting column.
Mobile phase:
HPLC buffer A and sample buffer = 97.5% water, 2.5% acetonitrile and 0.1% formic acid
HPLC buffer B =97.5% acetonitrile, 2.5% water and 0.1% formic acid

As peptides eluted they were subjected to electrospray ionization and then entered into an LTQ Velos ion-trap mass spectrometer (ThermoFisher, San Jose, CA). Peptides were detected, isolated, and fragmented to produce a tandem mass spectrum of specific fragment ions for each peptide. Peptide sequences (and hence protein identity) were determined by matching protein databases with the acquired fragmentation pattern by the software program, Sequest (ThermoFisher, San Jose, CA) [3]. All databases include a reversed version of all the sequences and the data was filtered to between a one and two percent peptide false discovery rate.

## Supplementary References


1. Shevchenko, A., Wilm, M., Vorm, O. & Mann, M. Mass spectrometric sequencing of proteins silver-stained polyacrylamide gels. *Anal Chem* **68**, 850–858 (1996).

2. Peng, J. & Gygi, S. P. Proteomics: the move to mixtures. *J Mass Spectrom* **36**, 1083–1091 (2001).

3. Eng, J. K., McCormack, A. L. & Yates, J. R. An approach to correlate tandem mass spectral data of peptides with amino acid sequences in a protein database. *J Am Soc Mass Spectrom* **5**, 976–989 (1994).
