# Supplementary material for: Secreted parasite Pin1 isomerase stabilizes host PKM2 to reprogram host cell metabolism
Source: Commun Biol. 2019 Apr 30;2:152. doi: 10.1038/s42003-019-0386-6 (PMC6491484; doi:10.1038/s42003-019-0386-6)
Supplement: Supplementary file 3 — Description of Additional Supplementary Files [file 42003_2019_386_MOESM3_ESM.docx]

**Description of Supplementary Data 1**

**File type: Excel.**

**List of proteins identified by Mass-Spectrometry using HA-Flag-purification.**

Double-affinity purification was performed using HA-Flag antibodies from NIH/3T3 control (empty pREV vector) and Flag-HA-hPin1 stable cell lines. Mass spectrometry identification of proteins immunoprecipitated in control cytoplasm and nuclear fractions and in Flag-HA-hPin1 cytoplasm and nuclear fractions are shown. Number of identified unique and total peptides, reference, gene symbol and MWT are indicated. One of the most abundant cytoplasmic Pin1-interactor was PKM2.
